# HBsAg glycan isomer predicts on-treatment HBsAg decline in low-HBsAg chronic hepatitis B on nucleos(t)ide therapy

**DOI:** 10.1186/s12985-026-03227-w

**Published:** 2026-06-19

**Authors:** Daisuke Yokoyama, Goki Suda, Masatsugu Ohara, Takatsugu Tanaka, Shoichi Kitano, Osamu Maehara, Ruixin Deng, Qingjie Fu, Zijian Yang, Naohiro Yasuura, Akimitsu Meno, Risako Kohya, Takashi Kitagataya, Naoki Kawagishi, Masato Nakai, Takuya Sho, Shunsuke Ohnishi, Naoya Sakamoto

**Affiliations:** 1 Department of Gastroenterology and Hepatology, Graduate School of Medicine, Hokkaido University, North 15, West 7, Kita- ku, Sapporo, 060-8638 Hokkaido Japan; 2 Laboratory of Molecular and Cellular Medicine, Faculty of Pharmaceutical Sciences, Hokkaido University, Sapporo, Japan

**Keywords:** Hepatitis B virus, Nucleos(t)ide Analog, HBcrAg, Entecavir, Tenofovir alafenamide, Tenofovir disoproxil fumarate, Retrospective, Multivariate, Decliners, Non-decliners

## Abstract

**Background:**

Early decline in HBsAg levels during nucleos(t)ide-analog (NA) therapy predicts subsequent HBsAg loss, but stratification tools are limited. We assessed whether a novel baseline HBV biomarker—the HBsAg glycan-isomer (HBsAgGi)—predicts 12-month HBsAg decline.

**Methods:**

We retrospectively analysed genotype C chronic hepatitis B patients who initiated entecavir, tenofovir-disoproxil-fumarate, or tenofovir-alafenamide and had serum available for HBsAgGi measurement. Early decliners were defined as those with ≥ 0.10 log10 IU/mL HBsAg reduction after 1 year. Baseline clinical and virological parameters were compared between decliners and non-decliners and within HBsAg < 3,000 and ≥ 3,000 IU/mL strata.

**Results:**

Of 201 screened individuals, 106 had samples for HBsAgGi testing. In this cohort, HBsAg levels continued to fall annually over 5 years in early decliners, whereas non-decliners showed almost no long-term HBsAg reduction. In univariate analyses, higher baseline HBsAg, HBV DNA, HBeAg-positivity, and HBcrAg were associated with 1-year HBsAg decline, whereas HBsAgGi was not; none remained significant in multivariable models. However, among patients with baseline HBsAg < 3,000 IU/mL, HBsAgGi alone differentiated decliners from non-decliners. After adjustment for baseline HBsAg and HBcrAg, lower HBsAgGi remained independently associated with ≥ 0.10 log10 IU/mL HBsAg decline at 1 year. In patients with baseline HBsAg ≥ 3,000 IU/mL, decliners had higher rates of HBeAg positivity and higher baseline HBsAg and HBcrAg. Traditional HBV markers were tightly correlated, whereas HBsAgGi showed only weak-to-modest correlations.

**Conclusions:**

Baseline HBsAgGi adds prognostic value in patients with low HBsAg, identifying those more likely to achieve on-therapy HBsAg decline and potentially informing selection for intensified or combination HBV therapies.

**Graphical abstract:**

**Figure:**
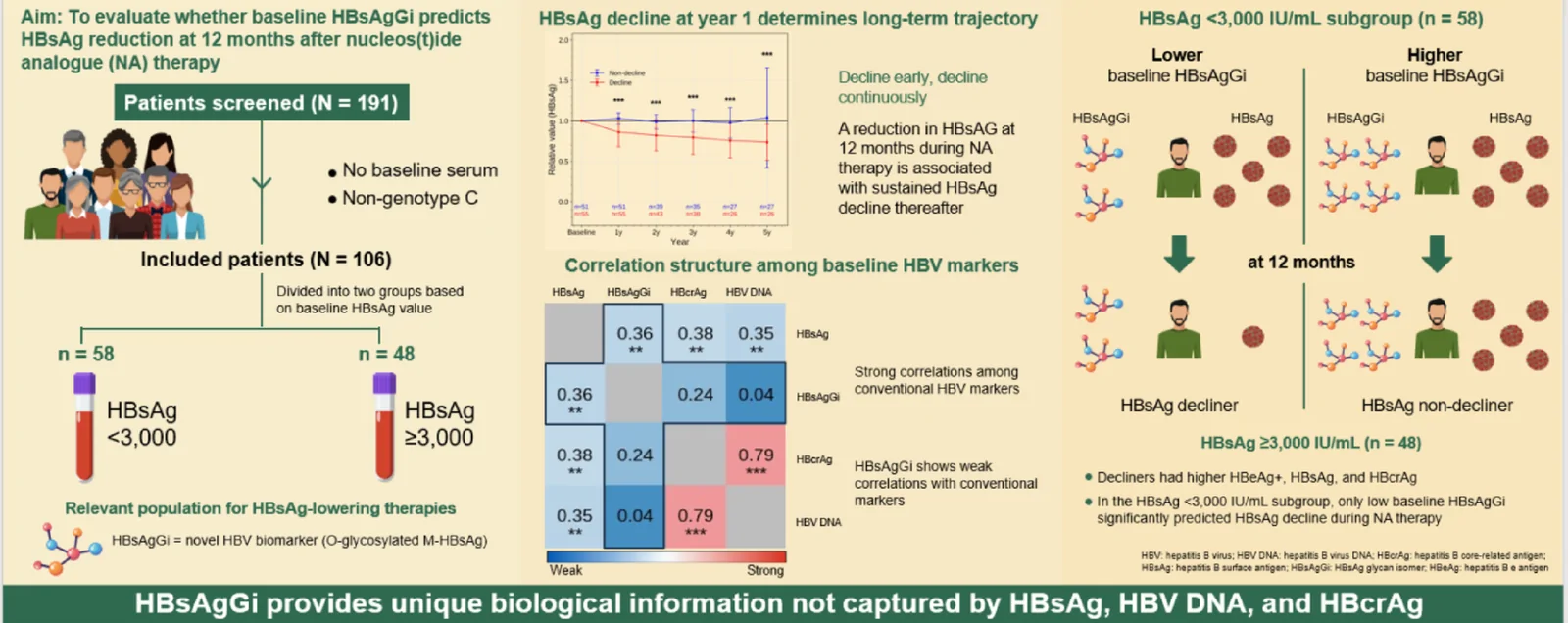

**Supplementary Information:**

The online version contains supplementary material available at https://doi.org/10.1186/s12985-026-03227-w.

## Background

Chronic hepatitis B (CHB) remains a major global health problem: in 2022, approximately 254 million people were living with chronic hepatitis B virus (HBV) infection worldwide, and approximately 1.1 million deaths were attributed to hepatitis B, predominantly from cirrhosis and hepatocellular carcinoma (HCC) [1].

Despite the near-universal suppression of viremia with long-term nucleos(t)ide analog (NA) therapy, sustained hepatitis B surface antigen (HBsAg) loss (functional cure) is rare, underscoring the need for early on-treatment risk stratification [2].

On-therapy HBsAg kinetics, particularly within the first treatment year, robustly predicts deeper serological endpoints, including HBsAg < 100 IU/mL and HBsAg loss, during continued NA therapy [3–5]. In particular, an early HBsAg decline within the first year of entecavir (ETV) or other NA therapies, especially in patients with low baseline HBsAg levels, is associated with a higher probability of subsequent HBsAg loss [3–5].

In addition to quantitative HBsAg, the HBsAg glycan isomer (HBsAgGi), an enzyme-linked immunosorbent assay (ELISA) signal that recognises O-glycosylated M-HBsAg enriched in infectious virions, enables a qualitative view of circulating HBsAg. HBsAgGi correlates with HBV DNA at baseline and with HBV RNA [6, 7]. Clinically, lower baseline HBsAgGi levels predict subsequent HBsAg seroclearance in patients with genotype C HBV infection and without NA treatment [8]. These data highlight HBsAgGi as a promising novel biomarker to complement the established serum markers in HBV management [2]. However, whether HBsAgGi can predict the on-treatment HBsAg decline during NA therapy remains unclear.

In parallel, antisense oligonucleotide (ASO) therapy has renewed attention to baseline HBsAg thresholds for patient selection. In the phase 2b B-CLEAR trial, bepirovirsen achieved sustained HBsAg and HBV DNA loss in a subset of participants, with higher response rates at lower baseline HBsAg [9]. Subsequent developments have emphasised a clinically actionable threshold of approximately 3,000 IU/mL, now reflected in ongoing phase 3 B-Well studies enrolling NA-treated adults with HBsAg levels > 100 to < 3,000 IU/mL [10, 11]. Moreover, in a randomised sequential bepirovirsen → peg-IFN study, all responders had baseline HBsAg levels < 3,000 IU/mL [9, 11].

In this study, we aimed to investigate whether HBsAgGi predicts HBsAg decline at 12 months after the initiation of NA therapy and whether its predictive performance differs between patients with baseline HBsAg levels < 3,000 and ≥ 3,000 IU/mL. These findings may support the use of HBsAgGi, particularly in patients with low baseline HBsAg (< 3,000 IU/mL), to help distinguish those who can be adequately managed with NA monotherapy from those in whom NA-based combination therapy with emerging agents, such as ASOs or small interfering RNA therapies [9], should be considered. In addition, we aimed to clarify the correlations among HBV markers, including HBsAgGi in the overall cohort and in subgroups with baseline HBsAg levels < 3,000 and ≥ 3,000 IU/mL.

## Methods

### Patients and study design

In this retrospective study, we screened patients with CHB or HBV-related liver cirrhosis who initiated NA therapy at Hokkaido University Hospital. Patients were included if they started NA therapy at Hokkaido University Hospital between September 2006 and May 2025, continued treatment for > 1 year, had complete clinical data, and had adequately preserved baseline serum samples available for HBsAgGi analysis. Patients were excluded if they had initiated NA prophylaxis to prevent HBV reactivation or reactivation-related hepatitis, had hepatic dysfunction corresponding to Child–Pugh class B or C, had coinfection with hepatitis C virus or human immunodeficiency virus, had poorly controlled cardiac disease, were receiving medications contraindicated with NA, had a history of liver transplantation, had received other anti-HBV agents, lacked adequately preserved serum samples, were aged < 20 years, or were infected with non-genotype C HBV. As HBsAg glycan isomer (HBsAgGi) has been validated primarily in patients with genotype C HBV infection, and the current assay system is applicable only to genotype C, we included only patients with genotype C HBV infection in this study. At NA initiation, the HBV infection status, including serum levels of HBsAg, HBcrAg, HBV DNA, HBeAg, and HBsAgGi, and clinical characteristics, including sex, age, aetiology of liver disease, blood counts, and standard biochemical parameters, such as alanine aminotransferase and aspartate aminotransferase levels, were evaluated. Subsequently, we analysed the factors associated with the decline in HBsAg levels 1 year after NA initiation. In addition, we evaluated the correlations among serum HBV markers, including HBsAgGi, in the overall cohort and in subgroups stratified according to baseline HBsAg levels < 3,000 and ≥ 3,000 IU/mL.

### Definition of hepatitis B surface antigen decline at 1 year after nucleos(t)ide analog initiation

Based on previous reports showing that serum HBsAg typically declines by approximately 0.1 log₁₀ IU/mL per year under long-term nucleos(t)ide analogue therapy [5, 12], and that a ≥ 0.3 log₁₀ IU/mL reduction over 3 years (≈ 0.1 log₁₀ per year) identifies patients with a higher probability of achieving low HBsAg levels and HBsAg seroclearance [5], we pragmatically defined early HBsAg decliners as those with a ≥ 0.10 log₁₀ IU/mL decline at year 1 after NA initiation.

Because this cutoff was selected a priori according to these previous reports [5], we examined whether early HBsAg decline defined by this threshold could predict sustained long-term reductions in HBsAg. To evaluate long-term on-treatment HBsAg kinetics, we calculated, for each group, the mean HBsAg level at yearly intervals up to year 5, expressed as a ratio to baseline (baseline set at 1.0), together with the standard deviation. Only patients with available HBsAg measurements at a given time point were included in the corresponding calculation, and the number of evaluable patients in each group at each year is shown as the number at risk in the figure.

### Tenofovir alafenamide and entecavir administration

Patients with chronic HBV infection received oral NA therapy as the standard of care at the discretion of the treating physicians. Tenofovir alafenamide (TAF) was administered at 25 mg once daily and ETV (0.5 mg) once daily. Tenofovir disoproxil fumarate (TDF) was orally administered at a dose of 300 mg daily. The selection of the agent and the timing of initiation followed routine clinical practice and guideline-based indications.

### Tests for hepatitis B virus markers

HBV-related assays were performed at a certified commercial laboratory (SRL, Tokyo, Japan), according to standard procedures, as previously described [13–17]. Serum HBsAg and total anti-HBc levels were measured using chemiluminescent immunoassays on ARCHITECT platforms (HBsAg-QT; Abbott Laboratories, Chicago, IL, USA). Serum HBV DNA was quantified using a real-time TaqMan polymerase chain reaction method (COBAS AmpliPrep/COBAS TaqMan HBV Test, version s2.0; Roche Diagnostics, Basel, Switzerland) with a reportable range of 1.3–8.3 log₁₀ IU/mL.

### Measurement of HBsAg glycan isomer by enzyme-linked immunosorbent assay

HBsAgGi was quantified using a commercial ELISA kit (RCMG Inc.), according to the manufacturer’s protocol [18]. Patient serum samples were immediately stored at − 20 °C after collection. The optical density was measured at 450 nm using a microplate reader. Concentrations were calculated from a standard curve corrected for any dilution factor. The reportable range of the assay was 10–200 ng/mL, and samples > 200 ng/mL were appropriately diluted and reanalysed to obtain values within this range.

### Statistical analyses

Continuous variables were assessed using the Mann–Whitney U test. Categorical variables were analysed using Fisher’s exact and chi-squared tests. Associations between two variables were evaluated using Spearman’s rank correlation coefficient. To explore the baseline factors associated with the 1-year on-treatment HBsAg decline, we first performed univariate analyses. Variables with a p value < 0.10 in univariate analyses were considered candidates for multivariate modelling. In addition, because quantitative HBsAg, HBsAgGi, and HBcrAg are all closely related to intrahepatic viral activity and are expected to be clinically relevant for the response to emerging HBsAg-lowering therapies, these three variables were included in the multivariate models, irrespective of their univariate p values. Given that a baseline HBsAg threshold of 3000 IU/mL has been proposed as a potential eligibility criterion for novel HBsAg-targeted agents, we a priori stratified patients into two subgroups (HBsAg < 3000 IU/mL and ≥ 3000 IU/mL). Multivariable logistic regression analysis was performed in the overall cohort and the subgroup with baseline HBsAg ≥ 3,000 IU/mL. In the subgroup with baseline HBsAg < 3,000 IU/mL, multivariable analysis was performed using Firth’s penalized likelihood logistic regression owing to the limited number of outcome events and the potential risk of small-sample bias. The same prespecified covariates (baseline HBsAg, HBsAgGi, and HBcrAg) were included in the penalized model, and penalized odds ratios, 95% confidence intervals, and p values were reported.

To assess the predictive performance of HBsAgGi in the subgroup with baseline HBsAg < 3,000 IU/mL, ROC curve analyses were performed and AUC, sensitivity, specificity, and the optimal cutoff value based on the Youden index were calculated. Model discrimination was compared between a reference model including baseline HBsAg and HBcrAg and an extended model additionally including HBsAgGi. Incremental predictive value was further assessed using continuous net reclassification improvement (NRI).

We assessed the correlations among HBV markers in the overall cohort and in subgroups with baseline HBsAg levels of < 3,000 and ≥ 3,000 IU/mL. To minimise multicollinearity, variables showing correlation coefficients of approximately ≥ 0.7 were not included in the same multivariate model.

All analyses were performed using IBM SPSS Statistics for Windows version 24.0 (IBM Japan, Tokyo, Japan) and the logistf package in R (version 1.26.1), as appropriate. Statistical significance was defined as two-sided p values of < 0.05.

## Results

### Patients

**Fig. 1:**
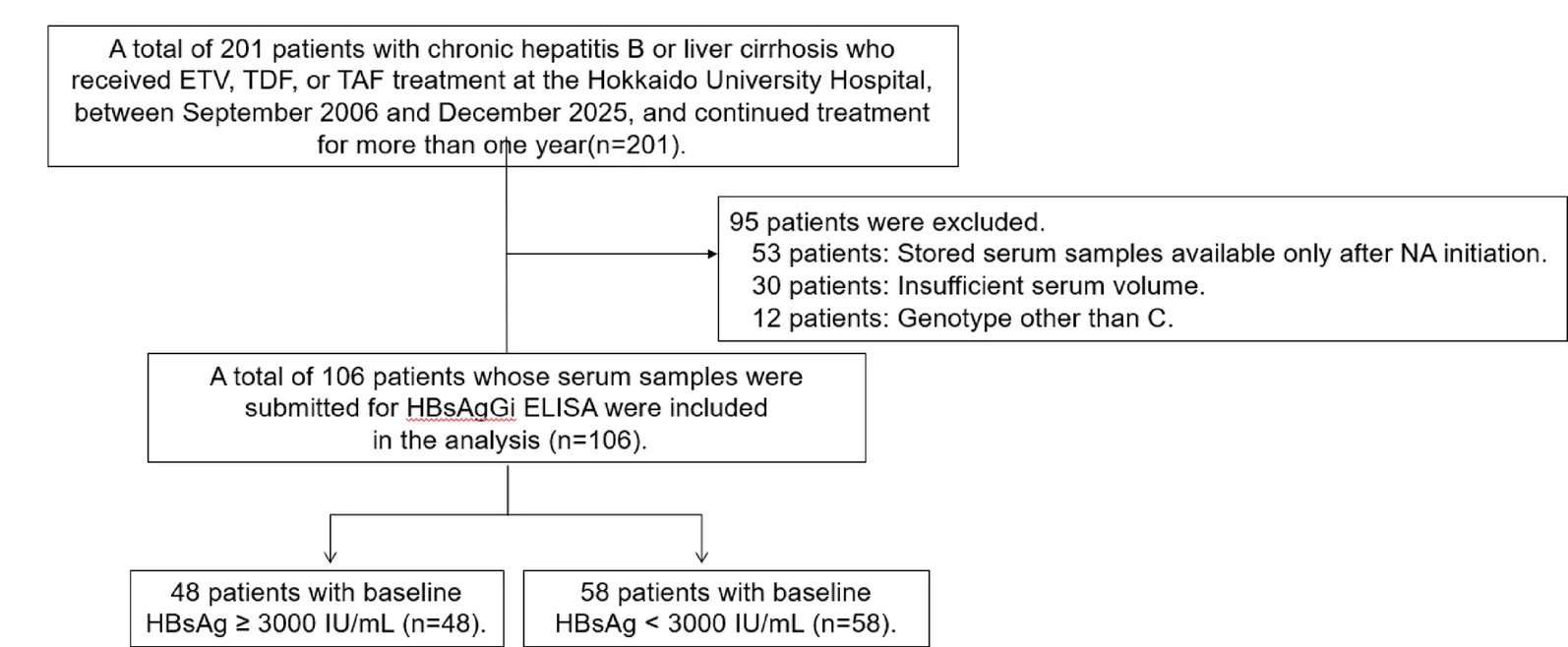
Study flowchart. HBV, hepatitis B virus; Na, nucleos(t)ide analog; TAF, tenofovir alafenamide; ETV, entecavir; eGFR, estimated glomerular filtration rate

Between September 2006 and May 2025, 201 patients with CHB or liver cirrhosis who initiated NA therapy with ETV, TDF, or TAF at Hokkaido University Hospital were screened and followed up ≥ 1 year. Among these, 106 patients had available baseline sera for HBsAgGi ELISA and comprised the analytic cohort; 95 were excluded because of a lack of baseline data (*n* = 83) or because the HBV genotype was non-C (*n* = 12). The final cohort included 106 patients with genotype C HBV infection who received NA (Fig. 1). At the start of NA, the median age of the 106 included patients was 54 years (interquartile range, 46–64 years), and 55.7% were male (Table 1).

**Table 1:** Baseline characteristics of the overall cohort and multivariate logistic regression results for HBsAg decline

Univariate analysis					Multivariate analysis					
Characteristics	All patients (*n* = 106)	HBsAg decline (*n* = 55)	HBsAg non-decline (*n* = 51)	*p* value	aOR (95% CI) Model A			*p* value	aOR (95% CI) Model B	*p* value
Age (years)	54 (46–64)	56 (44–63)	53 (47–66)	0.569	-			-	-	-
Male, n (%)	59 (55.7%)	34 (61.8%)	25 (49.0%)	0.241	-			-	-	-
Liver cirrhosis, n (%)	28 (26.4%)	14 (25.5%)	14 (27.5%)	0.829						
PLT×10⁴/µL	17.85(13.3–22.5)	17.9 (13.7–21.6)	17.5 (12.6–23.0)	0.754	-			-	-	-
Alb (g/dL)	4.0 (3.7–4.3)	4.0 (3.6–4.3)	4.1 (3.7–4.3)	0.628	-			-	-	-
AST (IU/L)	46 (33–77)	45 (33–80)	46 (35–71)	0.922	-			-	-	-
ALT (IU/L)	51 (33–110)	52 (32-109)	50 (34–109)	0.877	-			-	-	-
HBsAg (log IU/mL)	3.41 (2.98–3.93)	3.58 (2.98–4.46)	3.34 (2.96–3.52)	0.008	1.31 (0.84–2.05)			0.24	1.17 (0.73–1.89)	0.51
HBsAgGi (log ng/mL)	0.59 (0.38–0.81)	0.62 (0.36–1.23)	0.59 (0.41 − 0.80)	0.635	1.50 (0.68–3.35)			0.32	1.43 (0.64–3.18)	0.38
HBV DNA (log IU/mL)	6.55 (4.50–8.05)	7.40 (5.05–8.90)	5.60 (4.35–7.00)	0.002	-			-	1.16 (0.88–1.53)	0.3
HBeAg(+), n (%)	36 (34.0%)	24 (43.6%)	12 (23.5%)	0.040	1.68 (0.47–5.92)			0.43	1.16 (0.37–3.62)	0.79
HBcrAg (log U/mL)	5.10 (3.12–6.90)	5.90 (3.20–6.80)	4.40 (3.15–5.50)	0.048	0.99 (0.68–1.44)			0.95	-	-
NAs: ETV/TDF/TAF										
- ETV	71	33	38	0.094	1 (reference)			-	1(reference)	-
- TDF	8	7	1		4.71 (0.50–44.7)			0.18	5.25 (0.54–50.7)	0.15
- TAF	27	15	12		0.94 (0.34–2.60)			0.9	1.01 (0.36–2.85)	0.98

Data are presented as median (interquartile range, IQR) or number (%)

PLT, platelet count; Alb, albumin; ALT, alanine aminotransferase; AST, aspartate aminotransferase; HBsAg, hepatitis B surface antigen; HBsAgGi, O-glycosylated M-HBsAg; HBV, hepatitis B virus; HBeAg, hepatitis B e antigen; HBcrAg, hepatitis B core-related antigen; IQR, interquartile range; ETV, Entecavir; TDF, tenofovir disoproxil fumarate; TAF, tenofovir alafenamide fumarate; aOR, adjusted odds ratio; CI, confidence interval

### Comparison of patients with and without HBsAg decline at 1 year after NA initiation

**Fig. 2:**
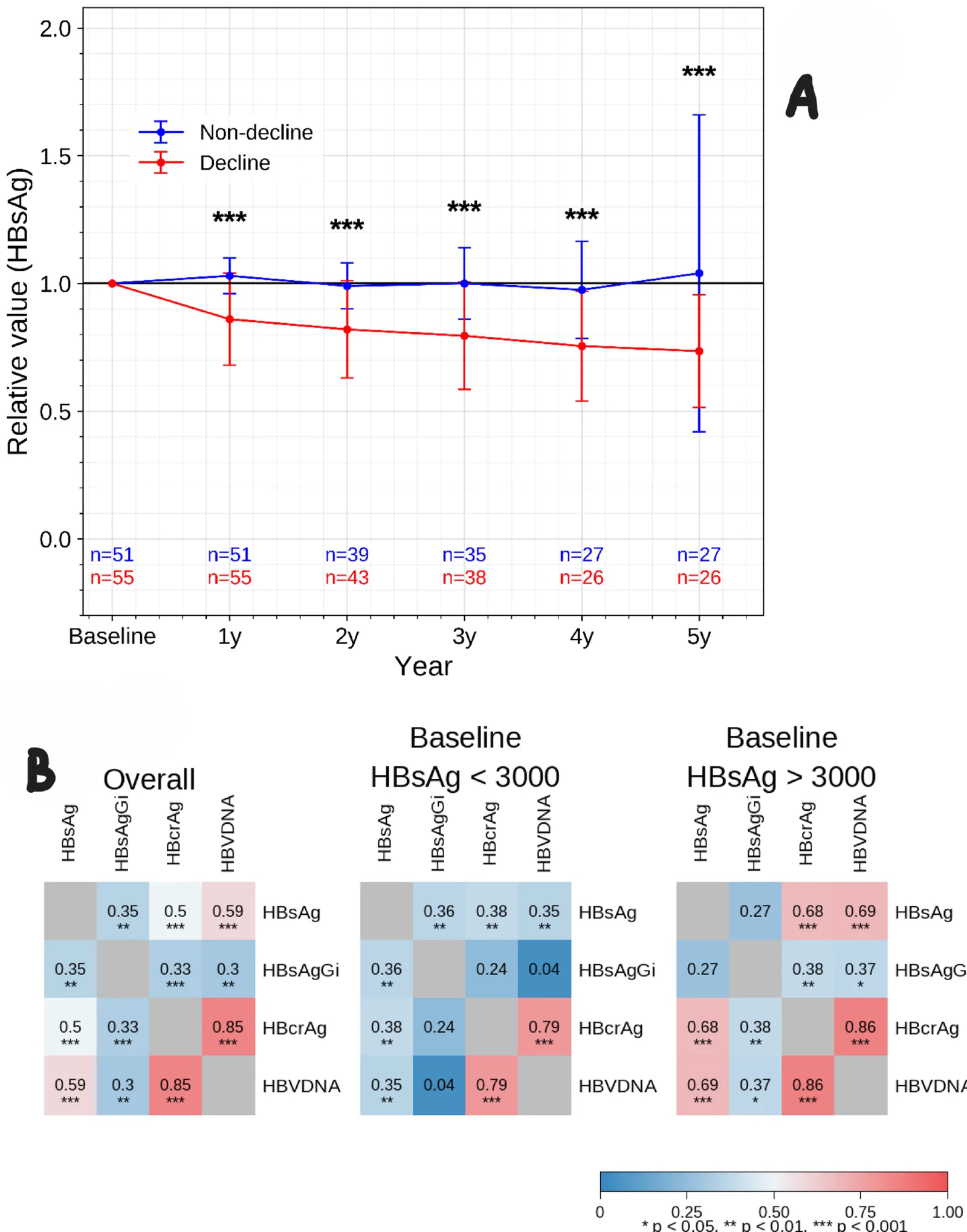
Comparison of long-term on-treatment HBsAg changes between patients with and without 1-year HBsAg decline and correlation structure among baseline HBV markers. **A.** Line graph showing, at yearly intervals over 5 years, the mean HBsAg level expressed as a ratio to baseline (baseline = 1.0) ± standard deviation in patients with and without 1-year on-treatment HBsAg decline. One-year HBsAg decline was defined as a reduction of ≥ 0.10 log₁₀ IU/mL in serum HBsAg levels at year 1 compared with baseline, and patients were accordingly classified into a decline and a non-decline group. Only patients with available HBsAg measurements at each time point were included in the corresponding mean values, and the numbers shown below the x-axis indicate the number of evaluable patients (“at risk”) in each group at each year. P values at each time point represent between-group comparisons of the HBsAg reduction ratio and were used to examine whether early HBsAg decline defined by this cutoff is associated with sustained long-term reductions in HBsAg. Data are presented as mean ± SD. *P < 0.05, ***P < 0.001. **B.** Correlation structure among baseline HBV markers in the overall cohort, patients with baseline HBsAg level < 3,000 IU/mL, and patients with baseline HBsAg level ≥ 3,000 IU/mL. Pairwise correlation coefficients among quantitative HBsAg, HBsAg glycan isomer (HBsAgGi), HBV DNA, and HBV core-related antigen (HBcrAg) levels are shown. The color scale represents the strength and direction of the correlation, with warmer colors indicating stronger positive correlations and cooler colors indicating weaker or negative correlations. HBV, hepatitis B virus; Na, nucleos(t)ide analog; TAF, tenofovir alafenamide; ETV, entecavir; eGFR, estimated glomerular filtration rate

Among the 106 patients, 55 (51.9%) met the predefined criterion for early HBsAg decline, achieving a ≥ 0.10 log₁₀ IU/mL reduction at 1 year after NA initiation. To evaluate long-term on-treatment HBsAg kinetics, we calculated, for each group, the mean HBsAg level at yearly intervals up to year 5. As shown in Fig. 2A, HBsAg levels in the decline group continued to decrease over the long term, whereas HBsAg levels in the non-decline group showed almost no reduction (at 5 years, decliners vs. non-decliners, *p* < 0.001) (Fig. 2A). These findings indicate that this prespecified cutoff value is suitable for predicting sustained on-treatment HBsAg decline during NA therapy. Patients with on-therapy HBsAg decline at month 12 entered treatment with higher baseline quantitative HBsAg (median, 3.58 vs. 3.34 log₁₀ IU/mL; *p* = 0.008) and HBV DNA (7.40 vs. 5.60 log₁₀ IU/mL, *p* = 0.002) compared to those without decline. They were also more frequently HBeAg-positive (43.6% vs. 23.5%, *p* = 0.040) and had higher baseline HBcrAg level (median, 5.90 vs. 4.40 log₁₀ U/mL; *p* = 0.048). In contrast, baseline HBsAgGi levels did not differ between the groups (0.62 vs. 0.59 log₁₀ ng/mL, *p* = 0.635). Although not statistically significant, the distribution of the NA types showed a trend toward an imbalance (*p* = 0.094). Other routine clinical and biochemical features were comparable between groups (Table 1).

In the overall cohort, multivariate logistic regression analyses did not identify any significant independent predictors of the 1-year HBsAg decline (Table 1).

### Comparison of patients with or without HBsAg decline at 48 weeks after NA initiation in patients with baseline HBsAg levels < 3,000 IU/mL

Among patients starting NA with lower HBsAg levels, only HBsAgGi differentiated subsequent HBsAg decline. Decliners exhibited lower baseline HBsAgGi levels than non-decliners (0.41 vs. 0.62 log₁₀ ng/mL, *p* = 0.014), whereas baseline HBsAg and HBV DNA levels were not significantly different between groups (Table 2).

**Table 2:** Baseline characteristics of the participants and multivariate logistic regression results for HBsAg decline in patients with HBsAg < 3,000 IU/mL

Univariate analysis					Multivariable Firth penalized logistic regression	
Characteristics	All patients (*n* = 58)	HBsAg decline (*n* = 24)	HBsAg non-decline (*n* = 34)	*p* value	Penalized OR (95% CI)	*p* value
Age (years)	58 (51–69)	60 (53–65)	57 (49–68)	0.538	-	-
Male, n (%)	34 (58.6%)	16 (66.7%)	18 (52.9%)	0.418	-	-
Liver cirrhosis, n (%)	22 (37.9%)	10 (41.7%)	12 (35.3%)	0.784		
PLT×10⁴/µL	16.8 (11.4–21.8)	17.6 (13.0–20.9)	15.5 (11.4–22.7)	0.856	-	-
Alb (g/dL)	4.1 (3.6–4.3)	4.1 (3.6–4.2)	4.1 (3.7–4.3)	0.956	-	-
AST (IU/L)	49 (33–80)	43 (32–83)	50 (38–75)	0.477	-	-
ALT (IU/L)	52 (35–115)	44 (29–94)	67 (42–115)	0.324	-	-
HBsAg (log IU/mL)	3.04 (2.12–3.26)	2.88 (2.28–3.16)	3.12 (2.12–3.34)	0.266	1.09 (0.63–1.95)	0.763
HBsAgGi (log ng/mL)	0.53 (0.36–0.68)	0.41 (0.19–0.59)	0.62 (0.44–0.78)	0.014	0.158 (0.02–0.90)	0.037
HBV DNA (log IU/mL)	5.10 (3.73–7.07)	5.10 (3.85–7.25)	5.10 (3.58–6.50)	0.608	-	-
HBeAg(+), n (%)	13 (22.4%)	4 (16.7%)	9 (26.5%)	0.526	-	-
HBcrAg (log U/mL)	4.15 (3.00-5.90)	3.95 (3.00–6.00)	4.20 (3.02–5.40)	0.557	1.01 (0.69–1.46)	0.970
NAs: ETV/TDF/TAF						
- ETV	45	18	27	0.538	-	-
- TDF	1	1	0		-	-
- TAF	12	5	7		-	-

Data are presented as Median (interquartile range, IQR) or number (%)

PLT, platelet count; Alb, albumin; ALT, alanine aminotransferase; AST, aspartate aminotransferase; HBsAg, hepatitis B surface antigen; HBsAgGi, O-glycosylated M-HBsAg; HBV, hepatitis B virus; HBeAg, hepatitis B e antigen; HBcrAg, hepatitis B core-related antigen; IQR, interquartile range; ETV, Entecavir; TDF, tenofovir disoproxil fumarate; TAF, tenofovir alafenamide fumarate; aOR, adjusted odds ratio; CI, confidence interval

We re-evaluated the multivariable analysis using Firth’s penalized likelihood logistic regression in patients with baseline HBsAg levels < 3,000 IU/mL, because only 24 decline events were observed in this subgroup. In this analysis, lower baseline HBsAgGi level remained independently associated with ≥ 0.1 log IU/mL HBsAg decline at 1 year after NA initiation (penalized OR, 0.158; 95% confidence interval [CI], 0.02–0.90; *p* = 0.037), whereas baseline HBsAg and HBcrAg levels were not significantly associated with the outcome (Table 2). Additionally, in sensitivity analysis additionally adjusting for NA type, lower baseline HBsAgGi remained independently associated with 1-year HBsAg decline (penalized OR 0.117, 95% CI 0.011–0.754, *p* = 0.021), whereas baseline HBsAg (OR 1.054, 95% CI 0.618–1.855, *p* = 0.849), HBcrAg (OR 1.029, 95% CI 0.707–1.495, *p* = 0.881), and NA type (tenofovir-based NA vs. ETV: OR 2.122, 95% CI 0.552–8.810, *p* = 0.274) were not significantly associated with the outcome (Supplementary Table S1).

In patients with baseline HBsAg < 3,000 IU/mL, baseline HBsAgGi showed moderate discriminatory ability for 1-year HBsAg decline, with an AUC of 0.691. The optimal cutoff value was 0.427 log10 ng/mL (~ 2.67 ng/mL), yielding a sensitivity of 58.3% and a specificity of 76.5%. When model-level discrimination was evaluated, the reference model including baseline HBsAg and HBcrAg showed an AUC of 0.544, whereas the extended model additionally including HBsAgGi showed an AUC of 0.705. Thus, addition of HBsAgGi improved model discrimination by 0.161. Continuous NRI for the extended model versus the reference model was 0.520, including an event NRI of 0.167 and a non-event NRI of 0.353, suggesting improved reclassification after addition of HBsAgGi (Supplementary Fig S1).

### Comparison of patients with or without HBsAg decline at 48 weeks after NA initiation in patients with baseline HBsAg level ≥ 3,000 IU/mL

In contrast, in patients with HBsAg level ≥ 3,000 IU/mL, multiple virological markers at baseline were associated with on-therapy HBsAg decline. Decliners had higher HBsAg (4.15 vs. 3.67 log₁₀ IU/mL, *p* = 0.001), HBV DNA (8.70 vs. 5.80 log₁₀ IU/mL, *p* = 0.002), HBsAgGi (1.16 vs. 0.54 log₁₀ ng/mL, *p* = 0.022), and HBcrAg (6.80 vs. 4.90 log₁₀ U/mL, *p* = 0.005) levels than non-decliners (Table 3).

**Table 3:** Baseline characteristics of the patients and multivariate logistic regression results for HBsAg decline in patients with HBsAg ≥ 3,000 IU/mL

Univariate analysis						Multivariate analysis											
Characteristics	HBsAg decline (*n* = 31)		HBsAg non-decline (*n* = 17)		*p* value	aOR (95% CI) Model A		*p* value	aOR (95% CI) Model B				*p* value	aOR (95% CI) Model C	*p* value	aOR (95% CI) Model D	*p* value
Age (years)	47 (38–61)		49 (47–54)		0.400	-		-	-				-	-	-	-	-
Male, n (%)	18 (58.1%)		7 (41.2%)		0.367	-		-	-				-	-	-	-	-
Liver cirrhosis, n (%)	4 (12.9%)		2 (11.8%)		1.000												
PLT×10⁴/µL	18.6 (14.3–24.7)		18.3 (16.5–23.2)		0.940	-		-	-				-	-	-	-	-
Alb (g/dL)	3.9 (3.7–4.3)		4.1 (3.9–4.2)		0.387	-		-	-				-	-	-	-	-
AST (IU/L)	48 (34–74)		38 (30–47)		0.155	-		-	-				-	-	-	-	-
ALT (IU/L)	59 (34–110)		37 (28–54)		0.108	-		-	-				-	-	-	-	-
HBsAg (log IU/mL)	4.15 (3.89–4.78)		3.67 (3.53–3.89)		0.001	22.1 (1.90–258)		0.01	-				-	-	-	-	-
HBsAgGi (log ng/mL)	1.16 (0.51–2.30)		0.54 (0.40–0.83)		0.022	5.21 (0.82–32.)		0.08	3.51 (1.04–11.9)				0.04	3.27 (1.01–10.60)	0.05	3.95 (0.95–16.5)	0.06
HBV DNA (log IU/mL)	8.70 (7.15–9.00)		5.80 (5.00–7.00)		0.002	-		-	-				-	1.26 (0.89–1.78)	0.2	-	-
HBeAg(+), n (%)	20 (64.5%)		3 (17.6%)		0.003	-		-	-				-	-	-	6.20 (1.31–29.4)	0.02
HBcrAg (log U/mL)	6.80 (5.15–7.00)		4.90 (3.90–5.60)		0.005	-		-	1.43 (0.92–2.24)				0.12	-	-	-	-
NAs: ETV/TDF/TAF																	
- ETV	15		11		0.458	-		-	-				-	-	-	-	-
- TDF	6		1			-		-	-				-	-	-	-	-
- TAF	10		5			-		-	-				-	-	-	-	-

Data are presented as medians (interquartile ranges) or numbers (%)

PLT, platelet count; Alb, albumin; ALT, alanine aminotransferase; AST, aspartate aminotransferase; HBsAg, hepatitis B surface antigen; HBsAgGi, O-glycosylated M-HBsAg; HBV, hepatitis B virus; HBeAg, hepatitis B e antigen; HBcrAg, hepatitis B core-related antigen; IQR, interquartile range; ETV, entecavir; TDF, tenofovir disoproxil fumarate; TAF, tenofovir alafenamide fumarate; aOR, adjusted odds ratio; CI, confidence interval

In the high-antigen stratum, multivariate logistic regression analysis revealed that HBsAg, HBcrAg, and HBeAg positivity were significantly associated with on-treatment HBsAg decline in some multivariate models (Table 3); however, these markers were strongly correlated with each other in the correlation analyses (Fig. 2B).

Among patients with baseline HBsAg ≥ 3,000 IU/mL, 10 of 48 patients achieved HBsAg < 3,000 IU/mL after 1 year of NA therapy. In univariate comparisons, baseline HBsAg level alone was significantly lower in those who achieved HBsAg < 3,000 IU/mL (Supplementary Table S2).

### Correlation structure among baseline HBV markers

Finally, we assessed pairwise correlations among baseline HBV markers in the overall cohort and in subgroups stratified according to baseline HBsAg levels < 3,000 and ≥ 3,000 IU/mL (Fig. 2B). In the overall cohort, conventional HBV markers (HBsAg, HBcrAg, and HBV DNA) showed moderate-to-strong intercorrelations, whereas HBsAgGi level consistently displayed lower correlation coefficients with each of these markers. A similar pattern was observed in both HBsAg strata. In patients with baseline HBsAg level ≥ 3,000 IU/mL, HBsAg, HBcrAg, and HBV DNA levels remained strongly intercorrelated, whereas their correlations with HBsAgGi level were clearly weaker. In patients with baseline HBsAg level < 3,000 IU/mL, conventional markers showed strong intercorrelations, whereas HBsAgGi level exhibited only weak or absent correlations with these markers, including an almost negligible correlation with HBV DNA.

## Discussion

The magnitude of HBsAg decline at 12 months after NA therapy has prognostic relevance; therefore, we used the 12-month time point for assessment in this study [4]. In our single-centre cohort (*n* = 106), we examined baseline factors associated with subsequent on-therapy HBsAg decline at NA initiation. In the univariate analyses of the overall cohort, higher baseline HBsAg, HBV DNA, HBeAg, and HBcrAg positivity were associated with a 1-year HBsAg decline, whereas HBsAgGi was not discriminative at the whole-cohort level (Table 1). However, when these variables were entered into multivariate logistic regression models, none remained independently associated with HBsAg decline. In contrast, among patients with baseline HBsAg level < 3,000 IU/mL, only HBsAgGi distinguished decliners from non-decliners in univariate analyses, with decliners exhibiting lower baseline HBsAgGi than non-decliners. Moreover, in Firth’s penalized likelihood logistic regression, a lower baseline HBsAgGi level remained independently associated with ≥ 0.1 log IU/mL HBsAg decline at 1 year after NA initiation (penalized OR, 0.158; 95% CI, 0.02–0.90; *p* = 0.037). Conversely, among patients with baseline HBsAg level ≥ 3,000 IU/mL, although HBsAg and HBcrAg levels were each significantly associated with on-treatment HBsAg decline in the high-antigen stratum, these markers were strongly correlated with each other, suggesting that they largely capture overlapping aspects of viral replication and antigen burden. Given that ongoing bepirovirsen programs are enrolling patients with HBsAg level < 3,000 IU/mL (e.g., B-Well-2, NCT05630820), these findings suggest that measuring HBsAgGi levels in the low-antigen range may help identify patients in whom additional HBsAg decline is unlikely with NA alone and for whom selective intervention with novel agents can be considered [9].

HBsAg loss is generally considered as an endpoint of “functional cure” and is associated with improved long-term outcomes, particularly a reduced risk of HCC, beyond mere suppression of viremia [19–21]. A low HBsAg level at NA initiation (e.g., < 1,000–3,000 IU/mL) and an early on-treatment decline in HBsAg are recognised predictors of the subsequent achievement of HBsAg level < 100 IU/mL and HBsAg loss [19]. In the present study, HBsAgGi may serve as a practical stratification marker in the low-HBsAg setting.

The threshold of 3,000 IU/mL is consistent with the inclusion criteria used in the phase 2 B-Clear trial and the phase 3 B-Well program of the ASO bepirovirsen [9]. This alignment facilitates external comparison with pivotal trials and supports the use of this threshold as a reasonable eligibility boundary for future novel therapies. In our stratified analyses, the incremental value of HBsAgGi was largely confined to this low-antigen group, supporting the clinical relevance of HBsAgGi measurements in patients who are candidates for bepirovirsen.

Moreover, in an NA-naïve genotype C CHB cohort with relatively low antigen levels (mean HBsAg of approximately 3,500 IU/mL), low HBsAgGi level was also a strong predictor of HBsAg seroclearance [8]. Ikeda et al. showed that HBsAgGi reflected the amount of infectious HBV virions in circulation and suggested that a higher virion burden might hinder HBsAg seroclearance [8]. Our findings in patients with baseline HBsAg level < 3,000 IU/mL are in line with these observations and indicate that in patients with relatively low HBsAg levels, low HBsAgGi levels may represent a robust biomarker of subsequent HBsAg loss or decline, irrespective of prior NA exposure.

A possible hypothesis for why HBsAgGi was associated with HBsAg decline only in the low-HBsAg group is that, in patients with relatively low baseline HBsAg levels, HBsAgGi may better reflect the virion-derived, cccDNA-related component of HBsAg production. In this setting, a low HBsAgGi level may indicate a lower ongoing cccDNA-dependent antigen production and a greater likelihood of further HBsAg decline during NA therapy. By contrast, in patients with higher baseline HBsAg levels, early HBsAg kinetics may be determined by the overall viral and antigen burden, which is already captured by conventional markers. This possible mechanism is consistent with previous reports on HBsAgGi and integrated HBV DNA [8, 22, 23].

Notably, the correlation among serum HBV markers in our cohort highlights the unique behaviour of HBsAgGi. HBV DNA level showed strong positive correlations with both quantitative HBsAg and HBcrAg levels, consistent with their shared reflection of intrahepatic viral replicative activity. In contrast, HBsAgGi level exhibited only a modest or even absent correlation with HBV DNA level, despite being enriched in infectious virions. This pattern suggests that HBsAgGi does not simply mirror the viral load but instead captures qualitative features of virion composition and envelope glycosylation that are not fully represented by conventional markers. Therefore, the relatively low correlation coefficients between HBsAgGi and the other HBV markers support the hypothesis that HBsAgGi provides largely independent biological information. Taken together with its significant association with on-treatment HBsAg decline, these findings indicate that HBsAgGi has the potential to serve as a novel and powerful biomarker, complementing established HBV markers rather than duplicating their roles.

In this study, 7 of 106 patients achieved HBsAg < 100 IU/mL within 5 years. Owing to the limited number of patients achieving these endpoints, no multivariable predictive analysis was performed. Baseline HBsAgGi appeared descriptively lower in patients who achieved HBsAg < 100 IU/mL (Supplementary Table S3). However, further investigation should be required.

From a mechanistic perspective, NA therapy rapidly suppresses reverse transcription and reduces HBV DNA, whereas total HBsAg generally declines only slowly, because circulating HBsAg represents a mixture of components: a fraction derived from infectious virions that are cccDNA-dependent and a fraction derived from subviral particles and integrated HBV DNA [22]. HBsAgGi, which targets O-glycosylated M-HBsAg, reflects the level of infectious virions and cccDNA-dependent particle production in the serum [6, 23]. Taken together, these findings suggest that in patients with baseline HBsAg level < 3,000 IU/mL and low HBsAgGi level, the proportion of virion-derived HBsAg is already small, and the cccDNA burden and transcriptional activity may be relatively suppressed, creating a milieu in which further HBsAg decline or even loss is more likely to occur. Confirmation of this hypothesis requires prospective studies involving paired intrahepatic assessments.

In contrast, among patients with high antigen levels (HBsAg level ≥ 3,000 IU/mL), those who showed a decline in HBsAg levels at 12 months had higher baseline HBsAg, HBV DNA, HBcrAg, and HBsAgGi levels than non-decliners. This pattern is consistent with the notion that patients with higher viral replication activity and stronger cccDNA-dependent antigen production have larger amounts of viruses and antigens that can be suppressed after NA initiation, leading to a more pronounced early decline in HBsAg levels [4, 23, 24]. Certainly, under NA therapy, HBsAgGi decreases in parallel with HBV DNA and RNA and behaves as a marker of replication burden [23]. From a clinical perspective, early on-treatment HBsAg kinetics in the high-antigen group appear to be driven mainly by the overall magnitude of viral and antigen loads and can be largely explained by conventional quantitative markers, such as HBsAg, HBV DNA, and HBcrAg. In this setting, HBsAgGi tends to track these quantitative load markers closely; therefore, its predictive value is limited. Nevertheless, it can be regarded as a complementary qualitative marker that reflects the cccDNA-dependent, replication-linked component of antigen production in patients with high HBsAg levels [4, 23, 24].

This study has some limitations. First, this was a single-centre, retrospective analysis, and paired intrahepatic data to assess cccDNA transcriptional activity and integration burden were not available. Second, the sample size was relatively small. Third, all patients had genotype C HBV infection, because HBsAgGi has been validated mainly in genotype C and the current assay is applicable only to this genotype. Therefore, the generalizability of our findings to other HBV genotypes remains uncertain. Additionally, the number of patients achieving HBsAg loss was too limited to reliably assess the predictive value of HBsAgGi for HBsAg loss. Furthermore, standardized assessment of hepatic steatosis was not available for all patients, and the potential influence of steatosis on HBsAg kinetics and HBsAgGi could not be fully evaluated. Larger prospective studies are required to confirm and extend the findings of this study. Nevertheless, this work highlights HBsAgGi, one of the newer HBV biomarkers, as a marker of previously unrecognised potential and should be considered a hypothesis generator, providing a basis for future investigations.

## Conclusion

Baseline HBsAgGi levels predict on-therapy HBsAg decline following NA treatment in patients with low baseline HBsAg levels (< 3,000 IU/mL). Incorporating HBsAgGi into the baseline assessment when the HBsAg level is < 3,000 IU/mL may improve risk stratification and guide optimal treatment selection.

## Supplementary Information


Supplementary material 1.


## Data Availability

All data generated or analysed during this study are included in this article. Further enquiries can be directed to the corresponding author.
