# Supplementary material for: HBsAg glycan isomer predicts on-treatment HBsAg decline in low-HBsAg chronic hepatitis B on nucleos(t)ide therapy
Source: Virol J. 2026 Jun 19;23:205. doi: 10.1186/s12985-026-03227-w (PMC13531837; doi:10.1186/s12985-026-03227-w)
Supplement: Supplementary file 1 [file 12985_2026_3227_MOESM1_ESM.docx]

**Supplementary Table S1.** Baseline characteristics of the participants and multivariate logistic regression results for HBsAg decline in patients with HBsAg < 3,000 IU/mL

| **Univariate analysis** | | | | | **Multivariable Firth penalized logistic regression** | |
| --- | --- | --- | --- | --- | --- | --- |
| **Characteristics** | **All patients (n=58)** | **HBsAg decline (n=24)** | **HBsAg non-decline (n=34)** | **p value** | **Penalized** **OR (95% CI)** | **p value** |
| Age (years) | 58 (51–69) | 60 (53–65) | 57 (49–68) | 0.538 | - | - |
| Male, n (%) | 34 (58.6%) | 16 (66.7%) | 18 (52.9%) | 0.418 | - | - |
| Liver cirrhosis, n (%) | 22 (37.9%) | 10 (41.7%) | 12 (35.3%) | 0.784 |  |  |
| PLT×10⁴/μL | 16.8 (11.4–21.8) | 17.6 (13.0–20.9) | 15.5 (11.4–22.7) | 0.856 | - | - |
| Alb (g/dL) | 4.1 (3.6–4.3) | 4.1 (3.6–4.2) | 4.1 (3.7–4.3) | 0.956 | - | - |
| AST (IU/L) | 49 (33–80) | 43 (32–83) | 50 (38–75) | 0.477 | - | - |
| ALT (IU/L) | 52 (35–115) | 44 (29–94) | 67 (42–115) | 0.324 | - | - |
| HBsAg (log IU/mL) | 3.04 (2.12–3.26) | 2.88 (2.28–3.16) | 3.12 (2.12–3.34) | 0.266 | 1.054  (0.618–1.885) | 0.849 |
| HBsAgGi (log ng/mL) | 0.53 (0.36–0.68) | 0.41 (0.19–0.59) | 0.62 (0.44–0.78) | 0.014 | 0.117  (0.011–0.754) | 0.021 |
| HBV DNA (log IU/mL) | 5.10 (3.73–7.07) | 5.10 (3.85–7.25) | 5.10 (3.58–6.50) | 0.608 | - | - |
| HBeAg(+),  n (%) | 13 (22.4%) | 4 (16.7%) | 9 (26.5%) | 0.526 | - | - |
| HBcrAg (log U/mL) | 4.15 (3.00-5.90) | 3.95 (3.00–6.00) | 4.20 (3.02–5.40) | 0.557 | 1.029  (0.707–1.495) | 0.881 |
| NAs: ETV/TDF/TAF |  |  |  |  | 2.122  (0.552–8.810) | 0.274 |
| - ETV | 45 | 18 | 27 | 0.755 | - | - |
| - TDF/TAF | 13 | 6 | 7 |  | - | - |

Data are presented as Median (interquartile range, IQR) or number (%).

Abbreviations: PLT, platelet count; Alb, albumin; ALT, alanine aminotransferase; AST, aspartate aminotransferase; HBsAg, hepatitis B surface antigen; HBsAgGi, O-glycosylated M-HBsAg; HBV, hepatitis B virus; HBeAg, hepatitis B e antigen; HBcrAg, hepatitis B core-related antigen; IQR, interquartile range; ETV, **Entecavir**; TDF, **tenofovir disoproxil fumarate**; TAF, **tenofovir alafenamide fumarate**; aOR, adjusted odds ratio; CI, confidence interval.

**Supplementary Table 2.** Baseline characteristics of patients with baseline HBsAg ≥3,000 IU/mL

according to HBsAg decline to <3,000 IU/mL after 1 year of NA therapy

|  | **Univariate analysis** | | |
| --- | --- | --- | --- |
| **Characteristics** | **<3000 (n=10)** | **>=3000 (n=38)** | **p value** |
| Age (years) | 51 (37–64) | 48 (42–59) | 0.889 |
| Male, n (%) | 8 (80.0%) | 17 (44.7%) | 0.075 |
| Liver cirrhosis, n (%) | 3 (30.0%) | 3 (7.9%) | 0.095 |
| PLT×10⁴/μL | 18.9 (13.6–23.6) | 18.5 (16.4–23.7) | 0.859 |
| Alb (g/dL) | 4.0 (3.8–4.2) | 4.0 (3.7–4.3) | 0.524 |
| AST (IU/L) | 45 (35–73) | 39 (32–68) | 0.657 |
| ALT (IU/L) | 56 (36–65) | 45 (28–81) | 0.648 |
| HBsAg (log IU/mL) | 3.57 (3.52–3.80) | 4.04 (3.76–4.71) | 0.008 |
| HBsAgGi (log ng/mL) | 0.81 (0.67–1.30) | 0.67 (0.40–1.23) | 0.388 |
| HBV DNA (log IU/mL) | 7.15 (5.18–8.32) | 7.75 (5.97–9.00) | 0.227 |
| HBeAg(+), n (%) | 3 (30.0%) | 20 (52.6%) | 0.292 |
| HBcrAg (log U/mL) | 4.95 (3.38–5.85) | 6.65 (4.68–7.00) | 0.077 |
| NAs: ETV/TDF/TAF |  |  |  |
| - ETV | 7 | 19 | 0.618 |
| - TDF | 2 | 13 |  |
| - TAF | 1 | 6 |  |

Data are presented as Median (interquartile range, IQR) or number (%).

Abbreviations: PLT, platelet count; Alb, albumin; ALT, alanine aminotransferase; AST, aspartate aminotransferase; HBsAg, hepatitis B surface antigen; HBsAgGi, O-glycosylated M-HBsAg; HBV, hepatitis B virus; HBeAg, hepatitis B e antigen; HBcrAg, hepatitis B core-related antigen; IQR, interquartile range; ETV, **Entecavir**; TDF, **tenofovir disoproxil fumarate**; TAF, **tenofovir alafenamide fumarate**; aOR, adjusted odds ratio; CI, confidence interval.

**Supplementary Table 3.** Baseline characteristics according to achievement of HBsAg <100 IU/mL within 5 years after initiation of NA therapy

|  | **Univariate analysis** | | | |
| --- | --- | --- | --- | --- |
| **Characteristics** | **All patients (n=106)** | **HBsAg < 100  (n=7)** | **HBsAg ≥ 100 (n=99)** | **p value** |
| Age (years) | 54.00 (46.00–64.75) | 61.00 (60.50–66.50) | 53.00 (45.00–64.50) | 0.022 |
| Male, n (%) | 59 (55.7%) | 6 (85.7%) | 53 (53.5%) | 0.129 |
| Liver cirrhosis, n (%) | 28 (26.4%) | 3 (42.9%) | 25 (25.3%) | 0.377 |
| PLT×10⁴/μL | 17.9 (13.3–22.5) | 13.6 (9.1–19.8) | 17.9 (13.4–22.6) | 0.252 |
| Alb (g/dL) | 4.0 (3.7–4.3) | 4.0 (3.6–4.3) | 4.0 (3.7–4.3) | 0.828 |
| AST (IU/L) | 46 (33–77) | 48 (35–78) | 45 (33–74) | 0.789 |
| ALT (IU/L) | 51 (33–110) | 67 (28–142) | 50 (34–105) | 0.804 |
| HBsAg (log IU/mL) | 3.41 (2.94–3.92) | 2.34 (2.13–2.68) | 3.46 (3.08–3.94) | 0.038 |
| HBsAgGi (log ng/mL) | 0.60 (0.37–0.82) | 0.30 (0.15–0.44) | 0.63 (0.40–0.83) | 0.042 |
| HBV DNA (log IU/mL) | 6.45 (4.40–7.88) | 7.50 (5.00–8.45) | 6.40 (4.40–7.80) | 0.422 |
| HBeAg(+),  n (%) | 36 (34.0%) | 3 (42.9%) | 33 (33.3%) | 0.687 |
| HBcrAg (log U/mL) | 5.10 (3.12–6.80) | 6.10 (4.15–6.90) | 5.10 (3.10–6.75) | 0.300 |
| NAs: ETV/TDF/TAF |  |  |  |  |
| - ETV | 71 (67.0%) | 5 (71.4%) | 66 (66.7%) | 0.059 |
| - TDF | 8 (7.5%) | 2 (28.6%) | 6 (6.1%) |  |
| - TAF | 27 (25.5%) | 0 (0.0%) | 27 (27.3%) |  |

Supplementary Figure S1

| **Model** | **AUC** | **Optimal cutoff** | **Sensitivity** | **Specificity** |
| --- | --- | --- | --- | --- |
| HBsAgGi alone | 0.691 | ≤0.427 log10 ng/mL | 58.3 | 76.5 |
| HBsAg + HBcrAg | 0.544 | ≥0.434 (predicted probability) | 41.7 | 76.5 |
| HBsAg + HBcrAg + HBsAgGi | 0.705 | ≥0.384 (predicted probability) | 75.0 | 61.8 |

|  |  |  |  |  |  |
| --- | --- | --- | --- | --- | --- |
|  |  |  |  |  |  |
|  |  |  |  |  |  |
|  |  |  |  |  |  |
